# Calves are socially motivated

**DOI:** 10.3168/jdsc.2021-0132

**Published:** 2021-10-22

**Authors:** Thomas Ede, Daniel M. Weary, Marina A.G. von Keyserlingk

**Affiliations:** Animal Welfare Program, Faculty of Land and Food Systems, University of British Columbia, Vancouver, BC, Canada V6T 1Z4

## Abstract

Most dairy calves are housed individually in the first weeks and sometimes months of their lives. Lack of social interaction can negatively impact feed intake, social skills, coping abilities, and cognitive performance, but the motivation of calves to seek companionship has seldom been investigated. In this study, 10 Holstein bull calves (*Bos taurus*; averaging 5.4 ± 2.6 d old upon entering the study) were housed individually in a central home pen with access to one pen on either side, each connected by a push gate. One side pen housed another calf of similar age and the same sex, and the second was otherwise identical in size and resources (feed and water) but without a social companion. Each time the test calf pushed open the gate to access a side pen, he would be left in it until the next feeding (approximately 0800 and 1600 h), at which time he was returned to the central home pen. After each successful pushing event, additional weight was added to the gate (initially a small amount, then incrementally higher). All calves but one pushed for the first time on d 1 of enrollment (within 9.4 ± 14.8 min of experimental start); the remaining calf pushed on d 3 of the test. Each calf was tested for 15 d and we recorded the maximum weight pushed for both side pens. Calves pushed a higher maximum weight (and pushed more frequently) for access to the pen with a social partner compared with the empty pen. We conclude that calves are socially motivated, even at a young age, and that calves can benefit from access to social contact.

## Highlights

• Calves pushed more weight to be with a social partner than alone.
• Calves learned an operant task without prior training.
• This research highlights the ability of calves to spontaneously display their social motivation.

## Graphical Abstract

**Figure:**
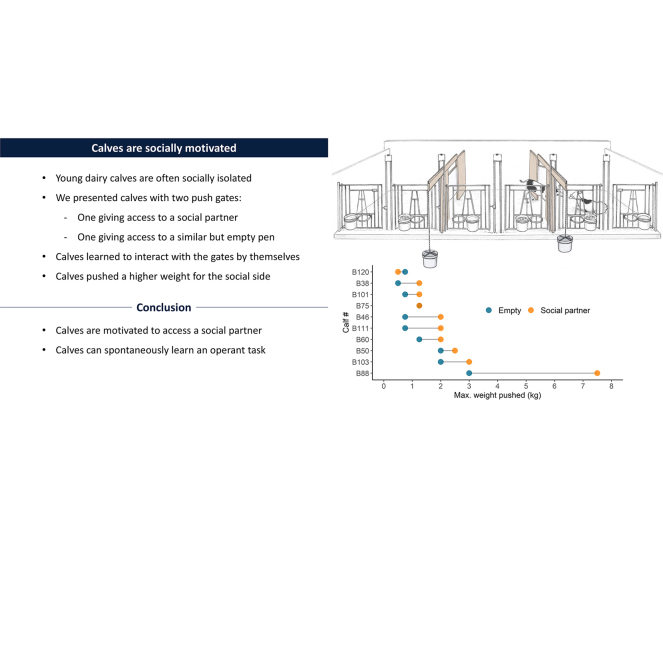
**Summary:** The objective of this study was to assess the motivation of young calves for a social partner. Calves were presented (without prior training) with two gates they could push, providing access either to a pen with a social partner or to a similar but empty pen. Every time calves pushed to enter one of the pens, additional weight was attached to its gate. Over 15 days, calves pushed a higher maximum weight for the pen with a social partner (compared with the empty pen). We conclude that calves are socially motivated and able to spontaneously display this motivation.

Most dairy calves are separated from the dam after birth and then reared individually for the first months of life. For example, in the United States and Europe, approximately 75% and 60%, respectively, of preweaning dairy calves are housed individually, typically in hutches or pens (Marcé et al., 2010; USDA, 2016). Individual housing allows for control of milk intake and prevents cross sucking. Some authors have suggested that individual housing also benefits calf health but evidence for this claim is limited (Jensen and Larsen, 2014; Costa et al., 2016).

Individual housing has known negative impacts on calves, including decreased solid feed intake, social skills, coping abilities, and cognitive performance (Costa et al., 2016; Bolt et al., 2017). Social separation (in the case of separation from their dam) induces a pessimistic cognitive bias (making calves less inclined to approach an ambiguous cue; Daros et al., 2014), and pair-housing induces an optimistic bias relative to single housing (Bučková et al., 2019). Early social housing has also been noted to affect feeding behaviors, with pair-housed calves showing increased solid feed intake through weaning and more frequent, smaller meals. Other authors have also demonstrated that early social contact induces a preference for feeding next to another calf (Miller-Cushon and DeVries, 2016).

Holm et al. (2002) trained calves using an operant conditioning task (calves learned to press a panel) to gain access to either full social contact with another calf or partial (head-to-head) contact. Conditioning required approximately 12 wk of training and data collection, so animals were tested at approximately 3 mo of age. Although this study provided evidence of social motivation in older calves, it did not address the first 6 to 8 wk of life when individual housing is most common.

Push gates have been used to assess motivation in older cattle, allowing an assessment of effort to access feed rewards (Franchi et al., 2019), roughage (Van Os et al., 2018), a mechanical brush (McConnachie et al., 2018), pasture (von Keyserlingk et al., 2017), a deep-bedded area (Tucker et al., 2018), and contact with their calf (Wenker et al., 2020). To our knowledge, push gates have not been used to assess motivation in calves.

The objective of this study was to assess the motivation of socially naive calves for contact with a conspecific. The push-gate task does not require training, instead relying upon spontaneous display of motivation. Calves were continuously presented with 2 push gates they could see and partially interact through: one providing access to an adjacent pen with a calf and the other leading to a pen physically identical (dimensions, feed, water) but without a social partner. Calves were observed for 15 d and, after every successful entry into one of the side pens, the weight on the respective gate was increased. We hypothesized that calves would be more motivated to access the pen with a social partner compared with the empty one, predicting a higher maximum weight pushed for this social side.

This study was approved by The University of British Columbia's Animal Care Committee, under the application A18-0198-A002.

Before enrollment, 20 Holstein bull calves (*Bos taurus*, experiment naive, 43.7 ± 5.7 kg birthweight) were housed in individual pens. The calves were blocked by pairs (n = 10). This sample size was calculated a priori to reach a power of 0.8, based on a paired *t*-test, with the difference of means equal to its standard deviation (https://www.r-project.org/). In each pair, one calf was assigned as the “subject” and the other as the “partner”; assignment between subject and partner was balanced by relative age of calf within the pair (i.e., the subject was the youngest for half the pairs). Age at enrollment averaged (±SD) 5.4 ± 2.6 d, with calves within pairs differing by 1.9 ± 3.8 d. Calves were bottle fed 4 L of whole milk twice daily (at approximately 0800 and 1600 h) and had ad libitum access to water, hay, and grain in buckets in front of the pens (Figure 1A).

**Figure 1:**
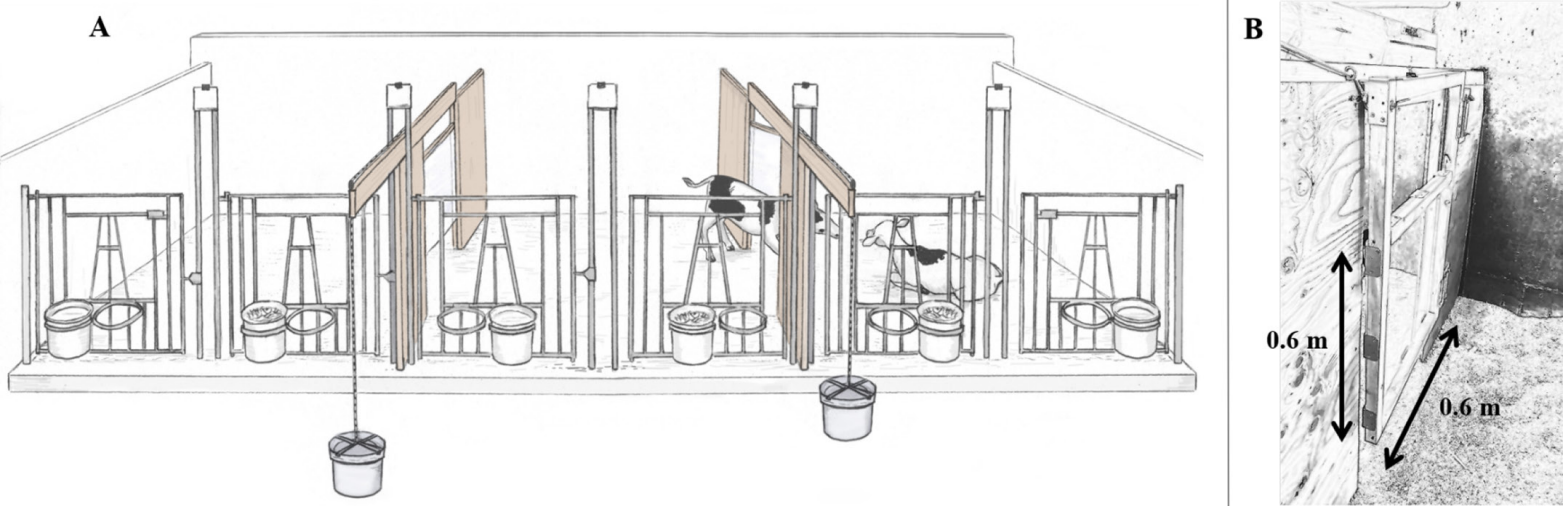
(A) Experimental apparatus. The “subject” calf in the middle has access to 2 side pens through a push gate: one with a social partner and one empty. Weight attached to the push gate is increased every time the subject accesses the pen. Illustration by Ann Sanderson (independent illustrator, Canada). (B) Close up of the push gate. Illustration made using Image to Sketch (https://imagetosketch.com/). Video of a calf pushing the gate: www.youtube.com/watch?v=36NEGndz0Xg.

The housing and testing apparatus measured 7.2 × 2.1 m and consisted of 3 pens (2.4 × 2.1 m) separated by one-way push-gates. Gates were 0.6- × 0.6-m plexiglass sheets with a wooden frame, connected to a rope that could be attached to a bucket and weighted. Transparent push gates were provided to facilitate a spontaneous understanding of where calves needed to push. Two 0.3-m gaps above and below the gate allowed calves partial physical contact (it was also possible for calves to touch while in separate pens through the holes giving access to buckets). Gates would swing closed after being pushed open, locking the subject into the side pen until the next milk feeding, at which time it was returned to the central pen. The subject was initially placed in the central pen, the partner was randomly allocated (by coin toss) to one of the side pens, and the other side pen was left empty. Placement of partner calves was balanced between sides (χ^2^ = 1.5, *P* > 0.05). The amount of water, grain, and hay in the buckets was the same in each of the 3 pens. Unfortunately, it was not possible to isolate the experimental apparatus because of space restrictions. Across from the experimental pen were individual pens in which calves were housed for up to 7 d. Previous research has noted the effect of the level of social contact on bonding between calves (Duve and Jensen, 2011), but we did not record the position of calves across from the experimental pen, and it is unknown whether they had an effect on the behavior of the subject calves.

For 15 d, calves were checked twice daily at the time of milk feeding (approximately 0800 and 1600 h). At that time, if the subject was in the middle pen, he was given the milk ration via bottle and not moved. If instead, the subject was in one of the adjacent pens (either with a partner or empty), he was returned to the middle pen, and the partner was randomly placed (by coin toss) in one of the adjacent pens; both calves were then fed their milk ration. If the subject calf had pushed for the partner, weight was added to the current pen with the partner; if the subject had pushed for the empty pen, weight was added to the current empty pen.

The weight attached to the gate was increased incrementally after each successful push, as detailed in Table 1. The use of a progressive ratio (Cooper et al., 2010) was based on our aim of using a task that was close to effortless for the calves at the beginning of the trial to facilitate learning, while allowing the detection of more substantial differences by the end of the 15-d trial. The initial 0.5 kg corresponded to the lightest bucket available, which would hold subsequent weights. The maximum weight pushed and number of pushes by each over the 15-d trial were recorded separately for the partner and empty pens, and the difference in these weights and number of pushes were tested using paired *t*-tests after checking graphically for normality. Correlations between birthweight and maximum weight pushed, number of pushes and experimental day, and average visit duration and maximum weight pushed were tested with a Pearson test using the cor.test function in R (https://www.r-project.org/).

**Table 1:** Increments of weight added to push gates after pushing events, in which the subject calf could push for either an empty pen or a pen with a social partner

Variable	Number of pushes													
	0 (start)	1	2	3	4	5	6	7	8	9	10	11	12	13
Weight added (kg)	0	0.50	0.25	0.25	0.25	0.25	0.50	0.50	0.50	0.50	1.0	1.0	1.0	—
Total weight (kg)	0	0.50	0.75	1.0	1.25	1.5	2	2.5	3.0	3.5	4.5	5.5	6.5	—

Of the 10 calves tested, 8 pushed more for the social side, 1 pushed more for the empty side, and 1 pushed the same weight for both sides (Figure 2A). Over the 15-d trial, calves pushed on average 1.0 kg more (with an average of 2.2 more pushing events) to access the social versus the empty pen [maximum weight: SD = 1.3 kg, 95% CI = (0.08, 2.0 kg), *t*_9_ = 2.5, *P* = 0.04; number of pushes: SD = 1.9, 95% CI = (0.9, 3.5), *t*_9_ = 3.7, *P* = 0.004]. One calf (B88) pushed more than twice the maximum weight pushed by any other calf. Removing this calf did not alter our statistical inferences [maximum weight: 95% CI = (0.2, 1.0 kg), *t*_8_ = 3.7, *P* < 0.01; number of pushes: 95% CI = (0.6, 3.2), *t*_8_ = 3.4, *P* = 0.01]. All calves but one opened a gate on the first day of testing (within 9.4 ± 14.8 min of entry into the apparatus); the remaining calf pushed open a gate on the third day. We found no effect of social partner on which pen was entered first (χ^2^ = 0.4, *P* = 0.5). On average, calves pushed open a gate 0.7 (±0.9) times per day (maximum possible was twice per day), taking 14.6 (±0.5) days, on average, to reach their maximum weight. No significant correlation between birthweight and maximum weight pushed was found [r = 0.4, 95% CI = (−0.3, 0.8), *t*_8_ = 1.3, *P* = 0.2]. Although calves had the highest number of pushes at the beginning and end of the trial (Figure 2B), we did not find a correlation between number of pushes and experimental day [social side: r = −0.04, 95% CI = (−0.5, 0.5), *t*_13_ = −0.1, *P* = 0.9; empty side: r = 0.3, 95% CI = (−0.3, 0.7), *t*_13_ = 1.0, *P* = 0.3]. Calves favored pushing in the 2 h following morning and evening feeding and, to a lesser extent, in the hours before evening feeding and at night (Figure 2C). Calves did not display a preference for pushing between morning and evening feeding or between evening and morning feeding (social side: χ^2^ = 0.5, *P* = 0.5; empty side: χ^2^ = 0.09, *P* = 0.8). Additionally, we did not find a difference in average duration of visits between the social and empty sides [social: 8.0 ± 2.3 h; empty: 9.6 ± 2.6 h; difference: 95% CI = (−4.0, 0.7), *t*_9_ = −1.6, *P* = 0.2] or a correlation between average duration of a visit and maximum weight pushed [social side: r = 0.4, 95% CI = (−0.3, 0.8), *t*_8_ = 1.4, *P* = 0.2; empty side: r = 0.2, 95% CI = (−0.5, 0.7), *t*_8_ = 0.4, *P* = 0.7].

**Figure 2:**
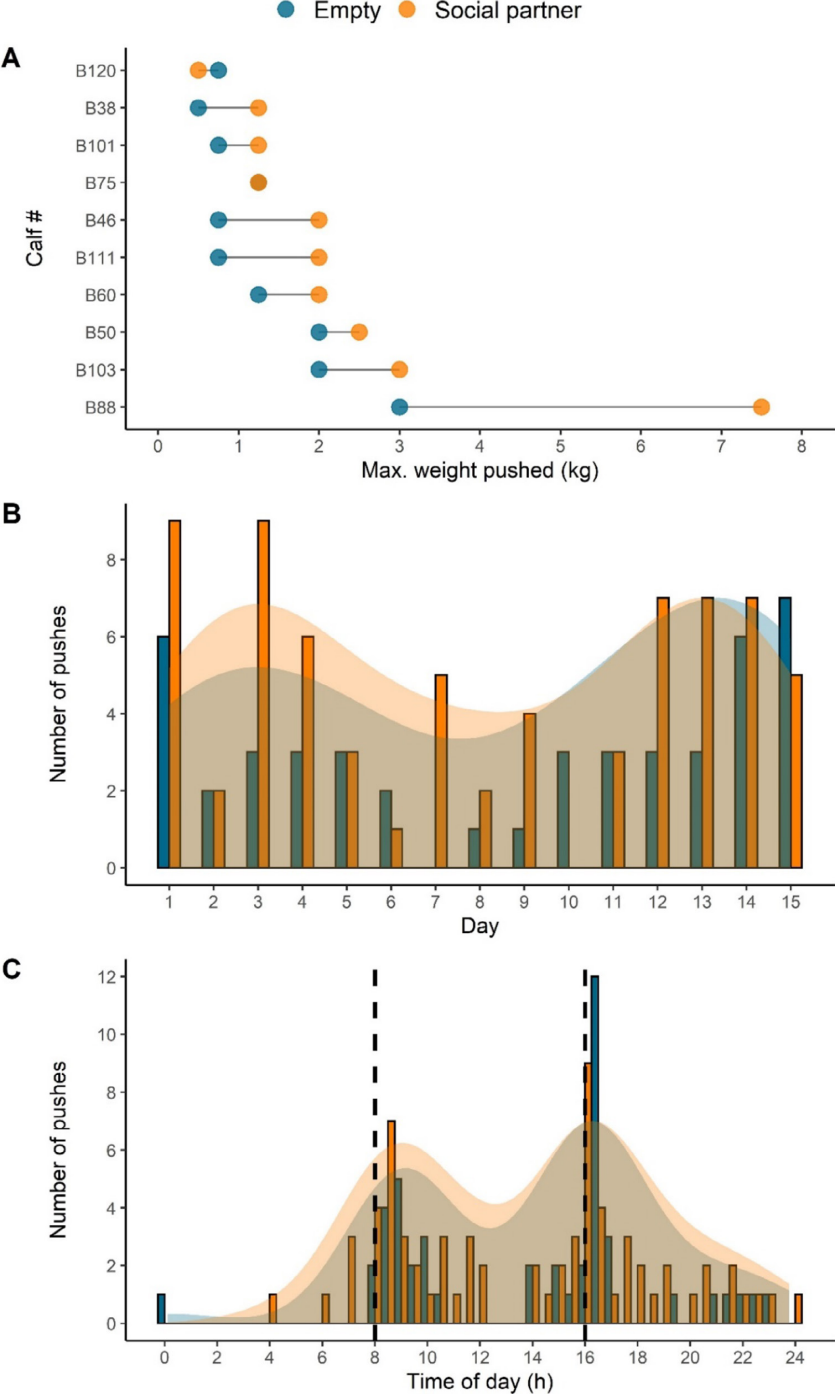
(A) Maximum weight pushed to open a push gate by each of 10 subject calves over the 15-d trial. Results are shown separately for the weight pushed to access the pen with another calf versus an otherwise identical but empty pen. (B) Distribution of pushes across the experimental days. (C) Distribution of pushes within days; vertical dashed lines represent feeding times. Shaded areas in panels B and C represent density estimates.

Preweaning dairy calves pushed more weight to access a pen with a social partner compared with an empty pen. This result indicates that young calves are motivated for full social contact, even when they have partial contact (calves could have visual and head-to-head contact with the second calf through the gaps above and below the push gate, even when closed). This result is in accordance those of Holm et al. (2002) for older calves. That even these younger calves are motivated for contact is in agreement with a study on free-ranging Maremma calves, showing that the time calves spent near their peers peaked at around 11 to 40 d of age (Vitale et al., 1986). Although previous research has reported a buildup in motivation for social play in calves (Bertelsen and Jensen, 2019), we did not find a correlation between pushes and experimental day, or a relationship between maximum weight pushed and reward length. This difference between studies could be due to the use of younger calves (<1 wk vs. >1 mo), longer reward times (several hours vs. 45 min), and smaller space allowance (5 m^2^ vs. 25 m^2^).

The testing method used in this study did not require training, but calves quickly learned to use the gate. This result suggests that pushing a see-through gate is sufficiently intuitive that calves can learn this quickly on their own, suggesting that this task could be used to efficiently assess motivation in other situations. Interestingly, all calves pushed for access to the empty pen to some degree. As the reward calf was positioned randomly between left and right, it may have been challenging for the subject calf to consistently identify the social pen, even with the see-through gate. We also suggest that calves were sufficiently curious (Meagher et al., 2017) to access the empty pen, especially when the push option was “cheap” (i.e., little effort was required). We limited the experimental period to 15 d, but as calves pushed their maximum weight during the last experimental day (or the day before), a longer experimental period could allow calves to express higher motivations. Moreover, longer term studies would allow the investigation of lasting effects of early social contact on calves, as noted previously for feeding patterns (Miller-Cushon and DeVries, 2016).

Calves displayed considerable variation in the maximum weight pushed (Figure 2A). Some of the subjects may have been more motivated to be with their partner; calves have been observed to display strong, specific social preferences (Raussi et al., 2010; Bolt et al., 2017; Lecorps et al., 2019), influenced by familiarity (Færevik et al., 2006; Duve and Jensen, 2011). In our study, calves only had access to a single, initially unfamiliar calf; future work should assess the strength of social preferences and the influence of age and familiarity on these preferences. Although no effect of birthweight was found and no difference in calf “technique” was noted, variation in the weight pushed may have been related to the strength of the calf or the calf's skill at opening the gate; assessing the weight pushed to access other resources would allow a better estimate of these individual differences. Pushes occurred more frequently around feeding times. We believe this is due to calves being most active and excited in these periods, and hence more likely to perform the physical task of pushing a gate. It is also possible that calves were not satiated and seeking additional milk, although it seems unlikely because subject calves did not receive milk in side pens.

A recent US survey found that approximately half the participants considered individual housing of calves unacceptable, often mentioning the lack of socialization as a major issue (Perttu et al., 2020). Our results add to the body of animal-based research highlighting the importance of social contact for calves (Costa et al., 2016). Calves displayed motivation for a social partner through an operant task without prior training, highlighting the potential of push gates to easily assess spontaneous animal motivation with limited human intervention.
